# Association Between Magnetic Resonance Imaging in Anesthetized Children and Hypothermia

**DOI:** 10.1097/pq9.0000000000000181

**Published:** 2019-05-23

**Authors:** Jessica A. Cronin, Christine Shen, Sohel Rana, Stanley Thomas Fricke, Andrew Matisoff

**Affiliations:** From the * Division of Anesthesiology, Pain and Perioperative Medicine, Children’s National Health System, Washington, D.C.; † Center for Surgical Care, Children’s National Health System, Washington, D.C.; ‡ Division of Diagnostic Imaging and Radiology, Children’s National Health System, Washington, D.C.

## Abstract

**Introduction:**

There is a myriad of factors that can lead to temperature derangements in anesthetized children undergoing magnetic resonance imaging (MRI). Temperature abnormalities in pediatric patients are associated with increased morbidity and mortality. Although some reports have looked at this topic, to our knowledge, no studies have continuously monitored temperature throughout the MRI scan. The purpose of this study is to determine the impact of MRI on body temperature for anesthetized children undergoing MRI using continuous temperature measurement, identify patient risk factors to develop temperature abnormalities, and determine the effect of temperature derangements on perianesthetic complications.

**Methods:**

This retrospective, single-center study evaluated 285 pediatric outpatients from January 1, 2018, to March 31, 2018, who were less than 8 years old and underwent anesthesia for an MRI scan. Temperature, postanesthesia care unit length of stay, and demographic data were collected retrospectively using chart review and data extraction from electronic medical records. Statistical analyses included unpaired *t* test, chi-square test, and simple and multiple linear regressions.

**Results:**

Sixty-three percent (179/285) of children in our study had a median temperature less than 36°C during their MRI scan. There were no patients who had a median temperature greater than 38°C during their MRI scan. There were no identifiable patient risk factors for the development of hypothermia. Those who developed hypothermia did not have an increased rate of perianesthetic complications.

**Conclusion:**

MRI in anesthetized children is associated with hypothermia but does not correlate with any significant perianesthetic complications.

## INTRODUCTION

The role of magnetic resonance imaging (MRI) is increasing for diagnosis and monitoring of medical conditions in children.^1^ In the setting of the guiding principle of ALARA (radiation dose “as low as reasonably achievable”) and the Image Gently campaign,^2^ MRI provides a potential alternative to computerized tomography (CT) and is a modality rich in diagnostic information.^3^ However, movement causes significant artifact and is a substantial barrier to high-quality images in this patient population. Children often require anesthesia to remain still in confined spaces for prolonged periods.

There are numerous risks associated with anesthesia for radiologic imaging including temperature derangements.^4,5^ Hypothermia (body temperature < 36°C) in pediatric patients increases the risk of metabolic acidosis, increased pulmonary and systemic vascular resistance, reduced cardiac output, increased oxygen consumption, and hypoventilation.^6^ In the adult population, perioperative hypothermia can increase the risk of developing surgical site infections, can alter blood coagulation, and can increase the length of hospital stay.^7^ In this setting, the Joint Commission and the Centers for Medicare and Medicaid Services have adopted normothermia-related quality metrics and performance on these metrics may influence reimbursement in the future.^8^

Some risk factors can lead to temperature derangements in anesthetized children undergoing MRI, including anesthesia, cool ambient temperatures, and radiofrequency radiation. Anesthesia is associated with hypothermia because it inactivates autoregulatory mechanisms such as vasoconstriction of peripheral afferents and reduces heat production. It also causes heat loss to the surrounding environment via radiation, convection, evaporation, and conduction.^7^ Neonates are at greater risk of hypothermia compared with older children and adults, as they have a higher ratio of surface area to body weight, reduced body fat, and an underdeveloped ability to shiver, which generates heat.^6^ The MRI environment is generally cool, approximately 18°C ± 3°C with low humidity. Heat loss to this surrounding cool environment can contribute to patient hypothermia.^9^ On the other hand, absorption of radiofrequency radiation from the MRI may increase the core temperature of the child.^10,11^

Some studies have looked at the impact of MRI on body temperature in children and infants with mixed results; some findings show hypothermia is frequent postscan.^9,12^ Other reports show hyperthermia (body temperature > 38°C) may be a bigger issue in this patient population.^10,11^ None of these previous studies have measured temperature continuously throughout the scan. Until recently, it was difficult to continuously measure temperature during MRI scans due to the lack of MR-compatible temperature probes. Temperature measurement in this setting was limited to temperature indicator strips which can be imprecise and are not visible to anesthesiologists or nurses during the scan. This project is the first quality initiative to characterize the association between MRI in anesthetized children and body temperature using continuous axillary temperature monitoring.

## METHODS

This project was a retrospective observational study. From January 1, 2018, to March 31, 2018, 441 outpatients less than 8 years old were anesthetized and then underwent an MRI scan of 1 or more body parts. We excluded 156 patients because they were febrile before the scan or temperature or postanesthesia care unit (PACU) data were not available. We also excluded patients who required additional procedures under the same anesthetic. Although this study was a retrospective review, it was a part of a Quality Improvement initiative looking at a temperature in pediatric patients undergoing MRI scans at Children’s National. Because the project did not fall into the category of human subject research, review by the Institutional Review Board was not required.

Temperature measurement followed standard of care at our institution. The trained nurse took a temperature with a temporal thermometer (TAT-5000; Exergen, Watertown, Mass.) prescan. After induction of anesthesia, transport to the scanner, and positioning of the patient in the scanner, the nurse placed a continuous fiber-optic temperature probe in the axilla. The automated electronic anesthesia record documented temperatures during this time every 5 minutes. Postscan, the recovery room nurse measured the patient’s temperature by the temporal thermometer. Induction of anesthesia generally involved mask induction with sevoflurane followed by maintenance of anesthesia with IV propofol. Nasal cannula provided oxygen supplementation, and some patients required oropharyngeal airways to preserve airway patency during the scan. Scanners used were Discovery MR450, Optima MR450W, and Discovery MR750 with DV26.X software (General Electric Healthcare Systems, Cleveland, OH). We obtained physiological data including temperature data using InVivo Expression MRI Patient monitor (Philips Medical/Invivo, Orlando, FL). Technologists at our institution keep the MRI fan on at the lowest of 3 settings for all scans unless the scanner is overheating or the anesthesiologist requests otherwise. Demographic data, body parts scanned, anesthesia time, scan time, heart rate data, and PACU time were all documented. We extracted these data from the electronic medical record through chart review and electronic data extraction. We excluded temperature measurements < 32°C as we believe these measurements reflected inappropriate temperature probe placement. We defined hypothermia as a median intrascan body temperature < 36°C and hyperthermia as median intrascan body temperature > 38°C. Efforts to reduce hypothermia in anesthetized children undergoing MRI during the study period were limited and nonstandardized.

Summary statistics were presented as means with standard deviations for continuous variables and as frequencies with percentages for discrete variables. Association of hypothermia with the length of anesthesia, age, sex, weight, heart rate, and the preoperative temperature was compared using unpaired *t* test and chi-square test, respectively, for continuous and categorical variables. Relationship of PACU duration with hypothermia, length of anesthesia, sex, age, propofol dose, preoperative temperature, and the postoperative temperature was explored using simple linear regression. Multiple linear regression was used to assess the relationship between PACU duration and hypothermia after adjusting for potential confounders including age, sex, weight, and anesthesia duration. Confounders were included in the regression model if they were significant at the 0.05 level or if they altered the coefficient of the primary predictor variable by more than 10%. *P* value of less than 0.05 was considered statistically significant and reported *P* values were 2-sided. We estimated the statistical power of our study to be 80% to detect an effect size of 0.35 for a 2-sided test at the level of alpha 0.05. We performed statistical analyses with Stata software, version 15.1 MP (Stata Corporation, College Station, Tex.).

## RESULTS

**Fig. 1.**
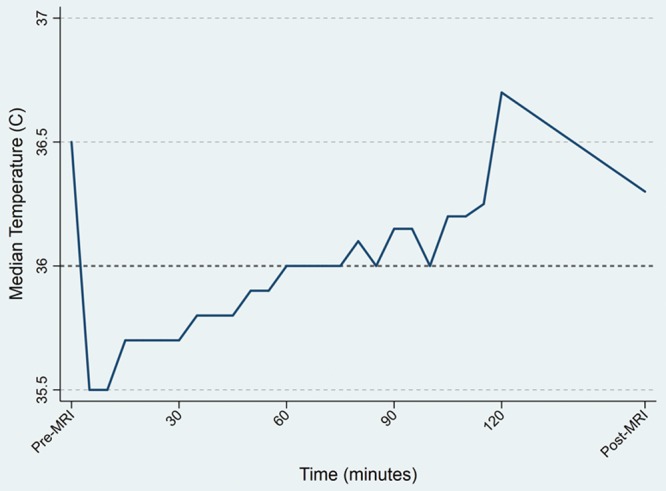
Median temperature over time.

The average age of the patients in the sample was 3.6 years (±2.1 years), 58.9% of whom were male. The median intrascan temperature was 35.7°C (±0.8°C). The incidence of intrascan hypothermia was 63% (179/285) (Table 1). There was no incidence of hyperthermia (0/285). Looking at the median temperature over time during the MRI scan, there was a visual trend toward a decrease in temperature with the initiation of anesthesia, followed by gradual warming throughout the scan (Fig. 1). The mean first intrascan temperature was 35.3°C (±1.1°C). There were no statistically significant associations between hypothermia and demographic information, anesthesia duration, or heart rate. There was no difference in length of PACU stay based on the hypothermic status of the patient, even adjusted for confounders. There was a statistical association between PACU length of stay and anesthesia duration, age, and weight (Table 2). Longer duration of anesthesia contributed to a longer PACU stay (*P* = 0.02). Age and weight were also positively associated with PACU length of stay (*P* = 0.004 and *P* ≤ 0.001, respectively). However, there were no significant associations between PACU length of stay and sex, propofol dose, temperature pre-MRI, or temperature post-MRI.

**Table 1.**
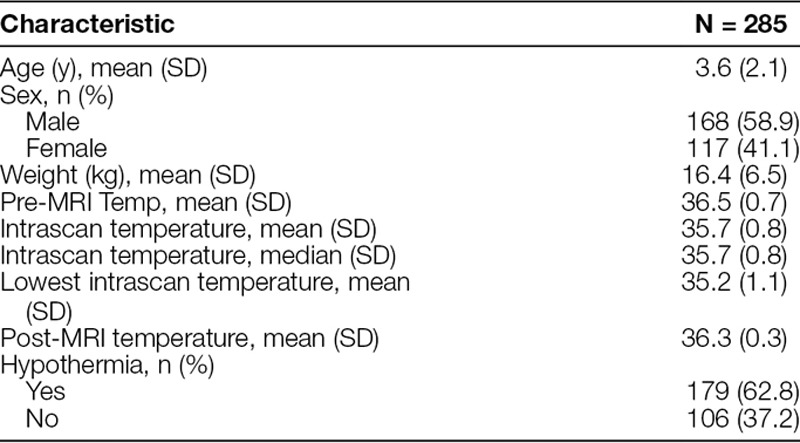
Demographics

**Table 2.**
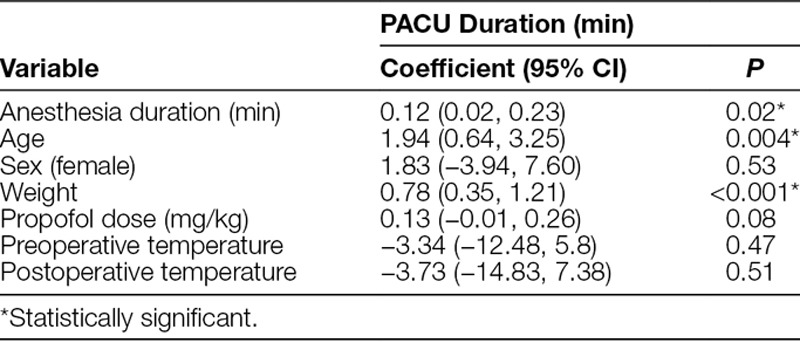
Association of PACU Length of Stay With Length of Anesthesia, Sex, Age, Propofol Dose, and Temperature Preoperatively

## DISCUSSION

In this study, we documented a 63% incidence of hypothermia (median temperature below 36°C) in children undergoing MRI scans with sedation. There was no association between hypothermia and age, weight, or duration of anesthesia. Those patients who became hypothermic had no increased length of PACU stay compared with normothermic children. Heart rate between the 2 groups was not significantly different. Larger, older children had a statistically significant increased length of PACU stay. Potential explanations for this include decreased the clearance of propofol in larger children due to drug deposition in fatty tissues and more rapid emergence in smaller children due to hunger and increased metabolic requirements. Although there is a statistical association between PACU stay and duration of anesthesia, the clinical implications are minimal given the small coefficient. No major periprocedural complications occurred in any of the patients. Despite the minimal measured harm caused by hypothermia in our study, the high incidence is a cause for concern.

Hypothermia has several detrimental physiologic effects. Cooling can induce nonshivering thermogenesis via the release of norepinephrine in brown fat, increasing metabolic rate and oxygen consumption up to 3 times.^13^ This metabolic derangement can inhibit growth and increase the risk of infection. In younger children with threatened oxygen delivery due to respiratory failure, congenital heart disease, and infection, increased oxygen consumption can result in tissue hypoxia and organ injury.^13^ Larger numbers of smaller patients with more disease burden require MRI scans to assess higher-risk disease states such as congenital heart disease, prematurity, and neurological disease. Many of these patients require abdominal or cardiac MRI scans which leave larger body surface areas exposed to heat loss. Other studies have shown that these smaller, sicker patients are at increased risk of developing significant, life-threatening complications as a result of hypothermia.^13^ The authors recommend implementing interventions which minimize heat loss during anesthesia induction and MRI scans.

Unfortunately, methods to reduce heat loss in patients during MRI scans are limited. Standard of care involves the application of warm blankets and other methods of insulation to reduce heat loss. There are no approved methods to actively warm patients during this period. MRI conditional chemical warmers such as the Transwarmer (COOPERSURGICAL, Trumbull, CT) which use an exothermic reaction to generate heat for up to 2 hours demonstrate safety and effectiveness in other clinical settings, but no data exist documenting their efficacy in reducing hypothermia during MRI scans.^14^ Also, they may cause image artifacts which might reduce image quality. MR-compatible incubators are useful in select patients, but due to increased cost (that can easily exceed $500,000) and their sometimes detrimental effect on image quality, cannot be generalized to most pediatric patients who require MRI scans.^15^ They also may be difficult to use in cases where patients require sedation for MRI scans due to limited access to the patient’s airway.

**Fig. 2.**
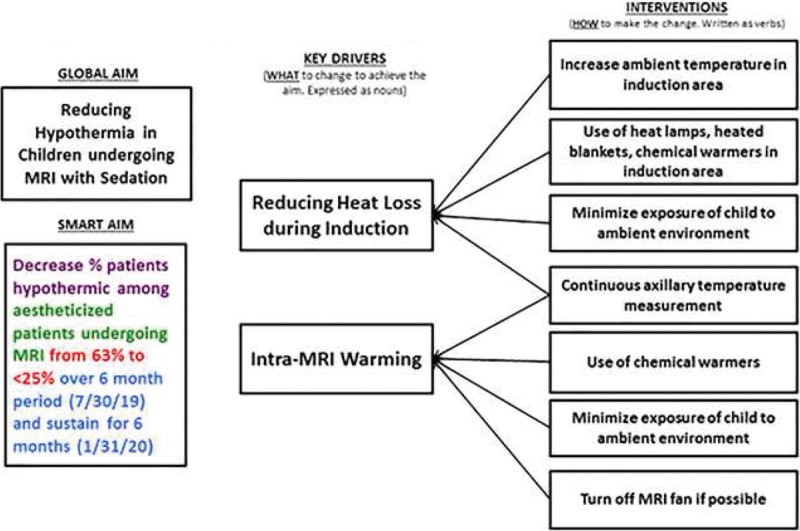
Key driver diagram.

Of note, many patients in this study became hypothermic after induction of anesthesia followed by gradually increasing temperatures during the scan. Typically, children who require anesthesia for MRI scans are induced under anesthesia in a designated area outside of the scanning environment and then moved to the scanning environment. The scanning environment is typically kept cool 18°C ± 3°C with a low humidity 40% ± 20%. Heat loss to the surrounding cool environment after induction of anesthesia, during transport into the scanning environment and upon arrival to the scanning environment likely result in the largest degree of heat loss documented in our study. Methods to actively warm patients during this period of significant heat loss may have the largest impact in reducing hypothermia. Therefore, as part of a quality initiative, these results indicate the potential for improvement in temperature management in these patients. Future initiatives will focus on reducing hypothermia, particularly during the anesthesia induction phase of peri-MRI scan period including the standardized use of chemical warmers, heated blankets, and heating lamps. These key interventions will reduce hypothermia in this population (Fig. 2). We believe an appropriate aim with these proposed interventions will be to decrease the incidence of hypothermia among anesthetized patients undergoing MRI scan from 63% to less than 25% in 6 months.

One limitation of this study is the use of continuous axillary temperature to measure temperature. Ideally, an esophageal measurement of core temperature is more accurate. Esophageal measurement is a relatively invasive method that is difficult to obtain in a sedated patient apart from patients requiring endotracheal intubation as part of their anesthetic. Although axillary temperature measurement is less invasive and easy to apply, there are concerns about its accuracy with 1 study showing a difference of *+*0.45°C compared with true core temperature.^15^ Although this may account for the increased incidence of hypothermia in our study, many neonatologists would define hypothermia as temperatures below 36.5°C, so using this definition would likely still result in a large incidence of hypothermia. Also, the axillary temperature is the most common method to measure temperature in neonatal units and is considered a standard of care. Another limitation of the study is that the MRI fan settings were not documented and may have affected intrascan temperature changes. Finally, although the majority of patients required only deep sedation with propofol for their MRI scan, some patients required higher levels of sedation, or even general anesthesia. Those patients who required general anesthesia for their scan may be at increased risk of heat loss compared with others. Other than looking at total propofol dose, we did not differentiate between the depth of anesthesia required during the study.

## CONCLUSIONS

This retrospective, single-center initiative demonstrated that a majority of children who underwent MRI scans with sedation in our study became hypothermic during their procedures. Those patients who developed hypothermia did not have an increased rate of perianesthesia complications nor the increased length of PACU stay. The study does highlight a potential concern in children who require MRI scans with sedation and are at higher risk of developing complications secondary to hypothermia. The authors recommend future research efforts looking at the effect of hypothermia during MRI scans on higher-risk patient populations and the development of methods to reduce the incidence of hypothermia during MRI scans.

## ACKNOWLEDGMENTS

The authors acknowledge the help of Dannette Spriggs.

## DISCLOSURE

The authors have no financial interest to declare in relation to the content of this article.
